# Extrasynaptic δGABAA receptors mediate resistance to migraine-like phenotype in rats

**DOI:** 10.1186/s10194-024-01777-4

**Published:** 2024-05-10

**Authors:** Berkay Alpay, Bariscan Cimen, Elif Akaydin, Filiz Onat, Hayrunnisa Bolay, Yildirim Sara

**Affiliations:** 1 Department of Medical Pharmacology, Faculty of Medicine, Hacettepe University, Sihhiye, Ankara, 06320 Türkiye; 2 Neuroscience and Neurotechnology Excellence Joint Application and Research Center (NÖROM), Ankara, 06560 Türkiye; 3 Department of Medical Pharmacology, Faculty of Medicine, Acibadem Mehmet Ali Aydinlar University, Istanbul, 34752 Türkiye; 4 Department of Neurology and Algology, Faculty of Medicine, Gazi University, Besevler, Ankara, 06560 Türkiye

**Keywords:** Migraine, GAERS, Inhibitory tonus, Mechanical allodynia, Light aversion, Extrasynaptic GABAA receptor

## Abstract

**Background:**

GABA, a key inhibitory neurotransmitter, has synaptic and extrasynaptic receptors on the postsynaptic neuron. Background GABA, which spills over from the synaptic cleft, acts on extrasynaptic delta subunit containing GABAA receptors. The role of extrasynaptic GABAergic input in migraine is unknown. We investigated the susceptibility to valid migraine-provoking substances with clinically relevant behavioral readouts in Genetic Absence Epilepsy of Rats Strasbourg (GAERS), in which the GABAergic tonus was altered. Subsequently, we screened relevant GABAergic mechanisms in Wistar rats by pharmacological means to identify the mechanisms.

**Methods:**

Wistar and GAERS rats were administered nitroglycerin (10 mg/kg) or levcromakalim (1 mg/kg). Mechanical allodynia and photophobia were assessed using von Frey monofilaments and a dark-light box. Effects of GAT-1 blocker tiagabine (5 mg/kg), GABAB receptor agonist baclofen (2 mg/kg), synaptic GABAA receptor agonist diazepam (1 mg/kg), extrasynaptic GABAA receptor agonists gaboxadol (4 mg/kg), and muscimol (0.75 mg/kg), T-type calcium channel blocker ethosuximide (100 mg/kg) or synaptic GABAA receptor antagonist flumazenil (15 mg/kg) on levcromakalim-induced migraine phenotype were screened.

**Results:**

Unlike Wistar rats, GAERS exhibited no reduction in mechanical pain thresholds or light aversion following nitroglycerin or levcromakalim injection. Ethosuximide did not reverse the resistant phenotype in GAERS, excluding the role of T-type calcium channel dysfunction in this phenomenon. Tiagabine prevented levcromakalim-induced mechanical allodynia in Wistar rats, suggesting a key role in enhanced GABA spillover. Baclofen did not alleviate mechanical allodynia. Diazepam failed to mitigate levcromakalim-induced migraine phenotype. Additionally, the resistant phenotype in GAERS was not affected by flumazenil. Extrasynaptic GABAA receptor agonists gaboxadol and muscimol inhibited periorbital allodynia in Wistar rats.

**Conclusion:**

Our study introduced a rat strain resistant to migraine-provoking agents and signified a critical involvement of extrasynaptic δGABAergic receptors. Extrasynaptic δ GABAA receptors, by mediating constant background inhibition on the excitability of neurons, stand as a novel drug target with a therapeutic potential in migraine.

**Graphical abstract:**

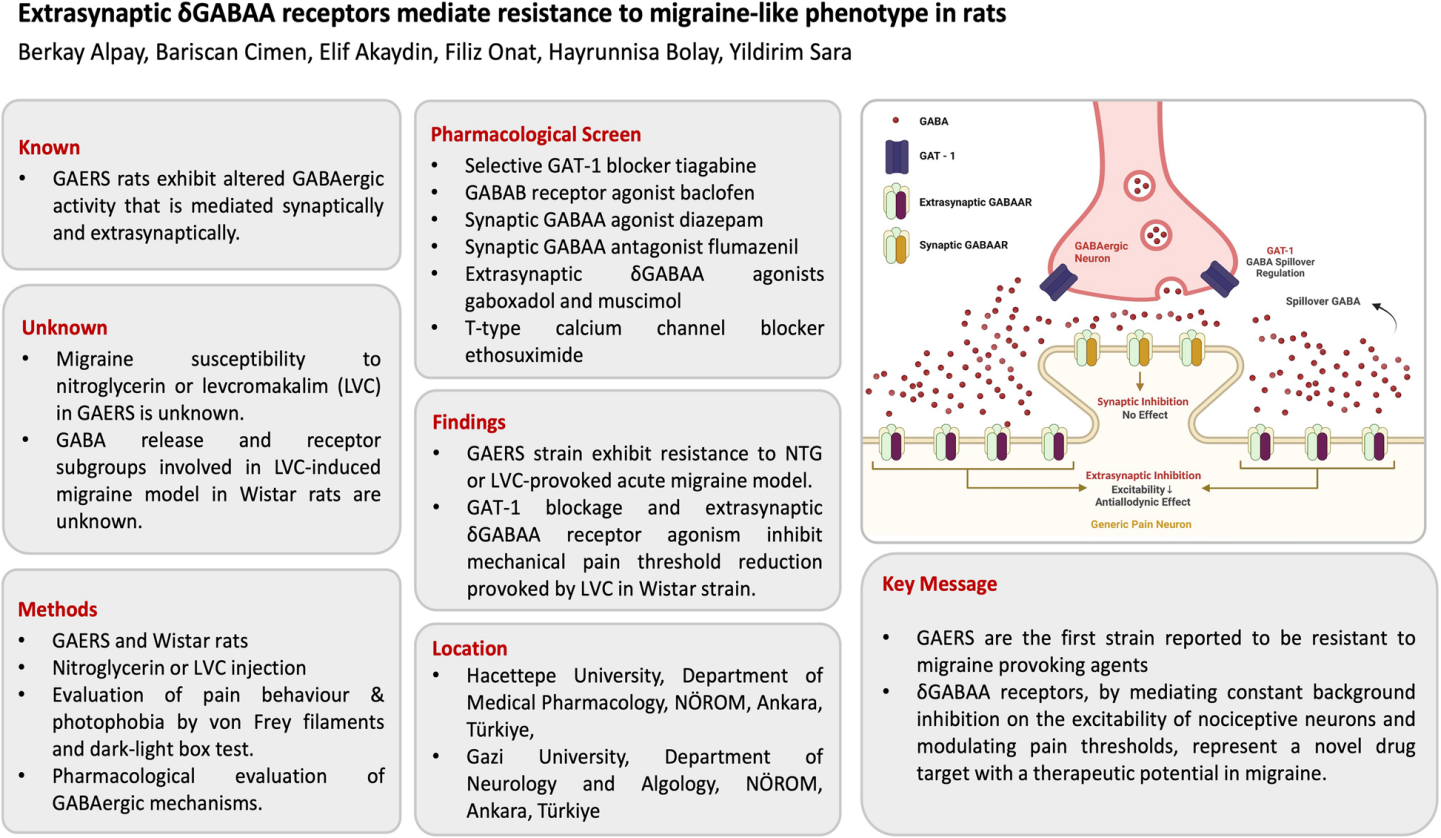

## Introduction

Migraine headache is a leading cause of morbidity among the adult population worldwide. Attacks are characterized by unilateral, pulsating, and moderate to severe headaches lasting 4-72 hours. Migraine attacks can be provoked in clinical studies by numerous agents such as nitroglycerin (NTG), levcromakalim (LVC), and calcitonin gene-related peptide (CGRP) [1, 2]. These agents were also translated into animal models to better study the pathophysiology of the disease. NTG injection was shown to reduce periorbital mechanical thresholds and provoke anxiety-like behavior and light aversion [3–5]. Similarly, LVC induced periorbital allodynia, anxiety-like behavior, and increased sensitivity to light [5]. The LVC-induced migraine-like phenotype was evident as early as 30 minutes following the injection and completely resolved after 120 minutes in contrast to NTG, which caused a longer-lasting phenotype. LVC can strongly cause vasodilation by mechanistically opening ATP-gated potassium channels and recruiting CGRP signaling [6, 7]. Previous research suggests that LVC primarily acts on meningeal vessels and adjoined peripheral afferents [8].

Gamma-aminobutyric acid (GABA) is the primary inhibitory neurotransmitter in the central nervous system. GABA’s inhibitory effects are mediated by two types of receptors, GABAA and GABAB receptors [9]. GABAA receptors are ligand-gated ion channels composed of 5 subunits. So far, 19 different subunit isoforms have been described: six α (alpha1-6), three β (beta1-3), three γ (gamma1-3), three ρ (rho1-3), and one each of the δ (delta), ε (epsilon), π (pi), and θ (theta) [10]. GABA decreases the likelihood of action potential generation and neuronal excitability through phasic and tonic inhibition modes [11]. Phasic inhibition is constituted mainly through synaptic GABAA receptors composed of αβγ subunits and are briefly activated upon presynaptic GABA release. The presence of γ subunit anchors the receptor to the postsynaptic membrane where GABA is directly released [12]. Tonic inhibition is mediated by “spillover” of GABA, a specific fraction of GABA that escapes the synaptic cleft, and acts on extrasynaptic GABAA and GABAB receptors [11, 13].

Extrasynaptic GABAA receptors have a particular configuration, harboring δ instead of γ subunit. The presence of δ subunit (and lack of γ subunit) in the GABAA receptor (δGABAA receptors) results in high affinity towards even low concentrations of ambient GABA and slow dissociation kinetics, allowing significant neuromodulation and provides a balance between excitation and inhibition [14–17]. Tonic inhibition decreases the size and duration of an excitatory postsynaptic potential, which narrows the temporal and spatial window for signal integration and reduces the likelihood of action potential generation [11]. δGABAA receptors are found ubiquitously in nearly all parts of the central nervous and trigeminal systems [11, 15, 18–22]. Previous studies have demonstrated the expression of δGABAA receptors in all neuroglial cells [23, 24]. The role of the GABAergic system has been implicated in migraine headaches [25–27]. Tonic inhibitory input may reduce the excitability of central pain pathways responsible for migraine pain propagation [27]. Any of the previous studies did not evaluate the involvement of extrasynaptic δGABAA receptors in migraine. It is not established if extrasynaptic δGABAA receptor activity is implicated in migraine phenotype.

Genetic Absence, Epilepsy of Rats Strasbourg (GAERS), is a particular strain of rats associated with increased GABAergic tonus and T-type calcium channel dysfunction [28, 29]. GABAergic excess is not due to increased vesicular GABAergic release or over-expression of extrasynaptic GABAergic receptors but from decreased reuptake of GABA in the synaptic cleft [30, 31]. GABAB receptors also play a significant role in the GAERS phenotype [32]. Nonetheless, the role of GABAA receptors is controversial. Recent evidence implies that alterations in phasic and tonic GABAA receptor-related activity contribute to the absence of epilepsy phenotype in a cell- and region-specific manner in the central nervous system [31]. In the study of Çarçak et al., the paired-pulse depression paradigm was more pronounced in GAERS rats than in normal Wistar rats [33]. It is uncertain whether this GABAergic excess can affect the susceptibility to migraine-inducing substances. A further question is which components of this GABA overactivity can contribute to a possible alteration of migraine-like phenotype susceptibility.

We hypothesize that GAERS have altered susceptibility to migraine-provoking agents due to two fundamental mechanisms, namely T-type calcium channels and GABAergic dysfunction. In this study, we showed that acute NTG or LVC injection do not provoke a migraine-like phenotype in GAERS rats, and we aim to establish 1) the contribution of T-type calcium channels to GAERS resistance, 2) the imitation of GAERS-like response by manipulating the GABA reuptake and GABA receptors in Wistar rats.

## Methods

### Animals

All animal experiments were approved by the Hacettepe University Animal Experimentation Ethics Board (approval no: 2022/05–11) in compliance with ARRIVE guidelines. Male Wistar and GAERS rats (250-350 g) were used throughout the experiments. Wistar rats were purchased from Kobay, Türkiye, and GAERS rats were obtained from the breeding colony of Acıbadem University, Türkiye. Rats were housed in triplets on a 12/12 h light/dark schedule (lights on at 07:00 h) in a climate-controlled room with ad libitum chow and water. Rats were acclimatized for at least one week before being subject to any experimental procedure.

### Experimental drugs and animal models of acute migraine

Acute migraine models are established by administering nitroglycerin (NTG – 10 mg/kg in 10% ethanol in saline) or levcromakalim (LVC – 1 mg/kg in 5% ethanol in saline) for a single time. Both drugs at the designated doses have been shown to induce an acute migraine-like phenotype in rats at the 120-minute and 60-minute time points, respectively [3, 5]. The vehicle for NTG and LVC in our experiments was ethanol in saline. All the information regarding experimental drugs and their administration is supplied in Table 1.

**Table 1:** Compounds used during the experiments

**Compound**	**Mechanism of Action**	**Dose**	**Concentration**	**Vehicle**
Nitroglycerin^a^	Nitric oxide donor	10 mg/kg IP	1 mg/ml	10% ethanol in saline
Levcromakalim^a^	ATP-gated potassium channel opener	1 mg/kg IP	0.1 mg/ml	5% ethanol in saline
Ethosuximide	T-type calcium channel blocker	100 mg/kg IP	25 mg/ml	Saline
Tiagabine	GABA reuptake inhibitor	5 mg/kg IP	5 mg/ml	Saline
Baclofen	GABAB receptor agonist	2 mg/kg IP	1 mg/ml	Saline
Diazepam	Synaptic GABAA receptor agonist	1 mg/kg IP	0.5 mg/ml	Saline
Flumazenil	Synaptic GABAA receptor antagonist	10 mg/kg SC	15 mg/ml	50% glycerol in saline
Gaboxadol	Extrasynaptic GABAA receptor agonist	4 mg/kg IP	4 mg/ml	Saline
Muscimol	Selective extrasynaptic GABAA receptor agonist	0.75 mg/kg IP	0.5 mg/ml	Saline

^a^Migraine-provoking substance

### Experimental design

For von Frey testing and pain behavior experiments, Wistar and GAERS rats were randomly divided into three treatment groups each, totaling up to 6 groups: control (CTRL), nitroglycerin (NTG), levcromakalim (LVC) for Wistar rat groups; a control group for GAERS (GAERS), GAERS nitroglycerin (GAERS NTG), GAERS levcromakalim (GAERS LVC) for GAERS rat groups. Control groups were injected with vehicle (ethanol in saline).

Periorbital and hind paw mechanical thresholds were measured following the injection of NTG, LVC, or vehicle. Baseline measurements were annotated as “BL” in the figures. In the relevant timeframes specified above (120 minutes for NTG, 60 minutes for LVC), light aversion was assessed in vehicle-, NTG-, and LVC-injected Wistar and GAERS rats. Subsequently, the possible therapeutic effects of other pharmacological interventions were evaluated with von Frey filaments. Only one experimenter conducted von Frey experiments and was blinded to animal condition.

### Determination of sample size

Sample sizes were determined by using G*power and resource equation [34, 35]. G-power analysis has retrieved nine animals per group for mechanical threshold testing and migraine-related light sensitivity experiment (α error: 0.05, β error: 0.8, effect size: 0.5). On the other hand, seven animals per group was calculated to be sufficient for both mechanical allodynia and light aversion tests from resource equation. Therefore, we have decided to use eight animals in each behavioral experiment. A total of 152 animals were used for all experiments.

#### Behavioral experiments

##### Mechanical allodynia

**Fig. 1:**
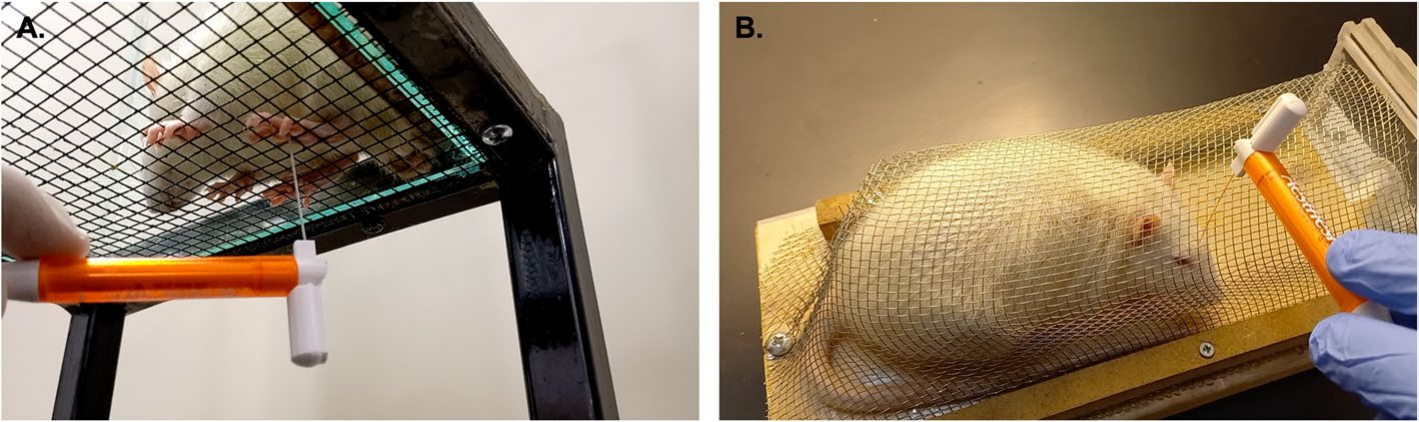
Illustration of the hind paw and periorbital sensory testing. **A** A rat is shown in the hind paw testing platform. Von Frey filament is applied to the mid-plantar area of the hind paw without touching the foot pads. **B** A rat is shown during periorbital testing in our apparatus, which allows rats to move and turn around. Von Frey filament is perpendicularly applied to the midline of the frontal area. A positive response was noted when the animal quickly withdrew its head from the filament

Von Frey monofilaments were used to measure periorbital and hind paw mechanical thresholds by the up-down method [36]. For hind paw threshold testing, rats were placed onto a flat wire mesh platform (Fig. 1A), and the experimental procedure was started after their exploratory behavior had ceased. Von Frey monofilaments were applied to the middle of the hind paw’s sole. Positive responses were characterized by paw withdrawal and paw licking [5, 37, 38] For periorbital testing, a large horizontal wire mesh tube apparatus (28 cm x 10 cm x 7 cm) was employed, as we used in our recent publications [5, 39] (Fig. 1B). The apparatus volume was five times larger than a standard restrainer device (15 cm x 4 cm x 6 cm) which allowed free movements without stress and animals never resisted to enter the apparatus in any of the trials. Animals were acclimated to handling and testing apparatus for five days (10 minutes per day) before the testing day [5, 39–41]. We began with a force of 2.0 grams and applied the filament perpendicularly to the midline of the forehead. Positive responses were identified as aversive head withdrawals, headshakes, vocalizations, ipsilateral head scratches, and whole-body retractions [42, 43]. To calculate the 50% withdrawal threshold, we utilized an online calculator accessible at https://bioapps.shinyapps.io/von_frey_app/ [44].

##### Light aversion

Light aversion was assessed using a dark-light box setup comprising two connected boxes: one brightly illuminated at 3000 lux and the other nearly completely dark [45]. Rats were allowed to explore both chambers freely during the test, and we recorded the duration they spent in the well-lit compartment and the count of transitions made from the dark area to the bright one over 15 minutes [46].

##### Statistical analysis

The results were not processed and were presented as raw data. The results are given as the mean ± standard error of the mean (SEM). Statistical analyses were conducted using GraphPad Prism software (GraphPad Software Inc., CA, USA). For data with a parametric distribution, we applied either Student’s t-test when comparing two groups or one-way or two-way analysis of variance (ANOVA) followed by the appropriate post hoc test when comparing three or more groups. Post hoc tests were not conducted in cases where the ANOVA F-values were not statistically significant. *P* < 0.05 was considered statistically significant.

## Results

### GAERS rats are resistant to acute mechanical allodynia induced by NTG or LVC injections

Ethanol in saline solutions was used as a vehicle in two different concentrations (5% and 10%) in our experiments. In each vehicle group, results from the von Frey testing and light aversion experiment were not statistically different; thereby, the results of the vehicle groups (5% and 10 % ethanol) were pooled. Wistar rats and GAERS rats had separate vehicle groups (*n*=8 per group).

**Fig. 2:**
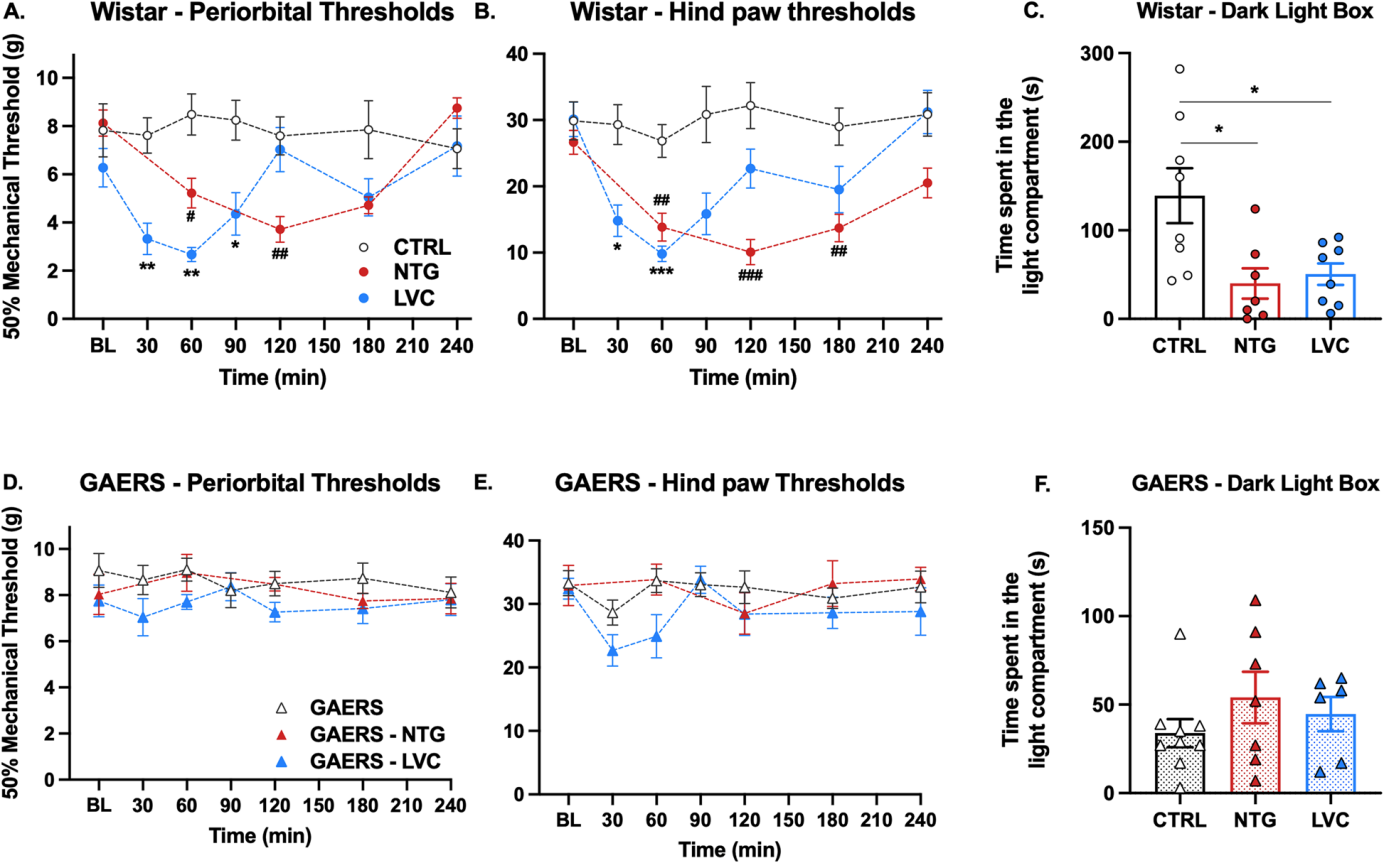
Acute LVC and NTG do not provoke migraine-like phenotype in GAERS. **A** LVC and NTG induced statistically significant periorbital allodynia (Sidak’s multiple comparisons two-way ANOVA, *P* < 0.05). **B** LVC and NTG injections caused mechanical threshold reduction in the hind paw plantar area (Sidak’s multiple comparisons two-way ANOVA, *P* < 0.01 and *P* < 0.001, respectively). **C** LVC and NTG-injected rats exhibited light aversion behavior as evident from less time spent in the light compartment of the dark light box test (Student’s t-test, *P* < 0.05). GAERS rats did not display periorbital (**D**) or hind paw (**E**) allodynia following LVC or NTG injection (Sidak’s multiple comparisons two-way ANOVA, *P* > 0.05). **F** GAERS rats did not show aversion to light after LVC or NTG administration (Student’s t-test, *P* > 0.05) (*n*=8 per group) (*P < 0.05 LVC vs CTRL; ** *P* < 0.01 LVC vs. CTRL; *** *P* < 0.001 LVC vs. CTRL; # *P* < 0.05 NTG vs. CTRL; ## *P* < 0.01 NTG vs. CTRL; ### *P* < 0.001 NTG vs. CTRL)

Firstly, we compared the allodynic effects of acute NTG or LVC injections to Wistar or GAERS rats with their corresponding control groups. Acute LVC significantly reduced periorbital thresholds in Wistar rats at the 30-,60- and 90-min timepoints (F (1, 15) = 8.16, *P* < 0.05, Fig. 2A). NTG-administered Wistar rats also exhibited statistically significant periorbital allodynia at the 60- and 120-min time points (F (1, 17) = 4.6, *P* < 0.05, Fig. 2A). However, periorbital mechanical thresholds of LVC- or NTG-injected GAERS rats were similar to GAERS at all timepoints (F (1, 15) = 3.11, *P* > 0,05 and F (1, 14) = 0.54, *P* > 0.05, respectively, Fig. 2D).

Similarly, hind paw mechanical thresholds of LVC group were detected significantly lower at the 30- and 60-min timepoints and NTG injection yielded an extracephalic allodynic response at 60-, 120-, 180-min timepoints in Wistar rats (F (1, 19) = 8.24, *P* < 0.01 and F (1, 23) = 21.99, *P* < 0.001, respectively, Fig. 2B). In contrast, GAERS LVC rats displayed a partial hind paw mechanical threshold reduction only at the 30-min timepoint, but this did not reach statistical significance (F (1, 15) = 4.02, *P* > 0.05, Fig. 2E). GAERS NTG group did not display any statistically significant threshold difference in the hind paw region (F (1, 14) = 0.0006, *P* > 0.05, Fig. 2E). We have also tested the effects of vehicle injection on the mechanical thresholds of GAERS rats and found no statistically significant difference at any timepoint.

### GAERS rats do not exhibit light aversion after NTG or LVC injections

We further conducted a dark-light box test to examine the resistance of GAERS phenotype to migraine-provoking substances. The behavioral testing was initiated when the lowest periorbital mechanical thresholds were detected (120 minutes for NTG and 30 minutes for LVC). GAERS rats underwent behavioral testing at these time points as well.

NTG- and LVC-injections provoked light aversion, as evident from less amount of time spent in the lightbox (*P* < 0.05, Fig. 2C). In contrast, GAERS NTG and GAERS LVC groups were not significantly different from GAERS (*P* > 0.05, Fig. 2F).

### Resistance of GAERS is independent of T-type calcium channel dysfunction

After verifying the GAERS resistance in both NTG- and LVC-induced acute migraine models, we continued our experiments with LVC. LVC was shown to provoke migraine attacks with a higher likelihood than NTG in clinical studies [47]. Although NTG is a more commonly used migraine-provoking substance, it has a broad and non-specific pharmacological target profile, some of which may be irrelevant to migraine pathogenesis, such as direct nitrergic action on the neuroglial system. On the other hand, LVC is an ATP-gated potassium channel opener, a more relevant and pathophysiologically better-characterized target concerning migraine phenotype [48]. Therefore, we decided that LVC was a better option for our study.

**Fig. 3:**
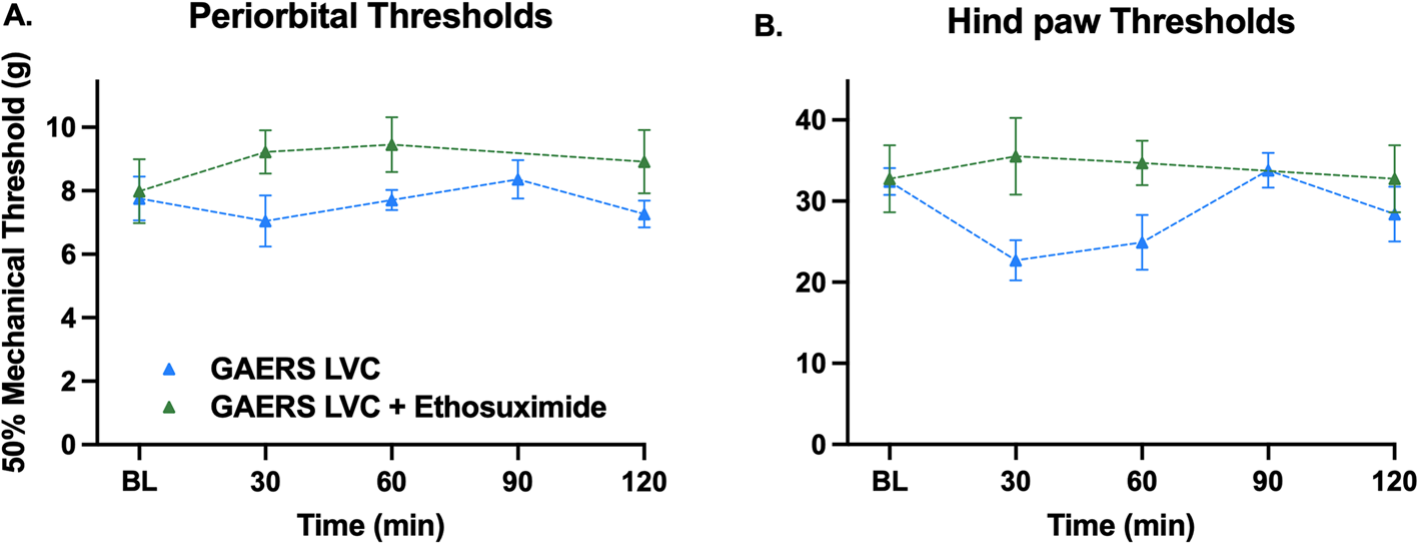
100 mg/kg ethosuximide does not reverse LVC-induced migraine-like phenotype resistance in GAERS. 100 mg/kg ethosuximide was administered to GAERS along with LVC to explore if T-type Ca channel overactivity would reverse the pain phenotype resistance in periorbital (**A**) and hind paw (**B**) areas. No change was detected at 30-min and 60-min time points; 90-min, 180-min, and 240-min tests were not performed (Sidak’s multiple comparisons two-way ANOVA, *P* > 0.05, *n* = 8 per group)

At first, we wanted to demonstrate if this resistance was caused by increased T-type calcium channel conductance. We blocked these channels by administering ethosuximide (100 mg/kg). GAERS rats, when injected LVC together with 100 mg/kg of ethosuximide, still had comparable mechanical thresholds with the GAERS group and did not display cephalic or extracephalic pain sensitivity (F (1, 9) = 3.26, *P* > 0.05, Fig. 3A and F (1, 9) = 4.79, *P* > 0.05, Fig. 3B respectively). As we did not detect any difference at 30-min and 60-min, we did not test further for 180- and 240-min time points.

Thus, it is inferred that the resistance of GAERS was not due to the T-type calcium channel dysfunction.

After observing that GAERS resistance is not a direct/indirect result of T-type calcium channel dysfunction, we decided to investigate whether the GABAergic system perturbations cause the pain-resistance phenotype. As stated above, dysfunctions in the GABAergic system are speculated to be trifold: 1) GAT-1 underactivity, 2) GABAB receptor overactivity, and 3) GABAA receptor dysfunction. In the following experiments, we imitated these dysfunctions in the normal Wistar rats to better grasp the mechanism of migraine-like pain resistance.

### GAT-1 antagonist tiagabine blocks mechanical allodynia in Wistar rats

**Fig. 4:**
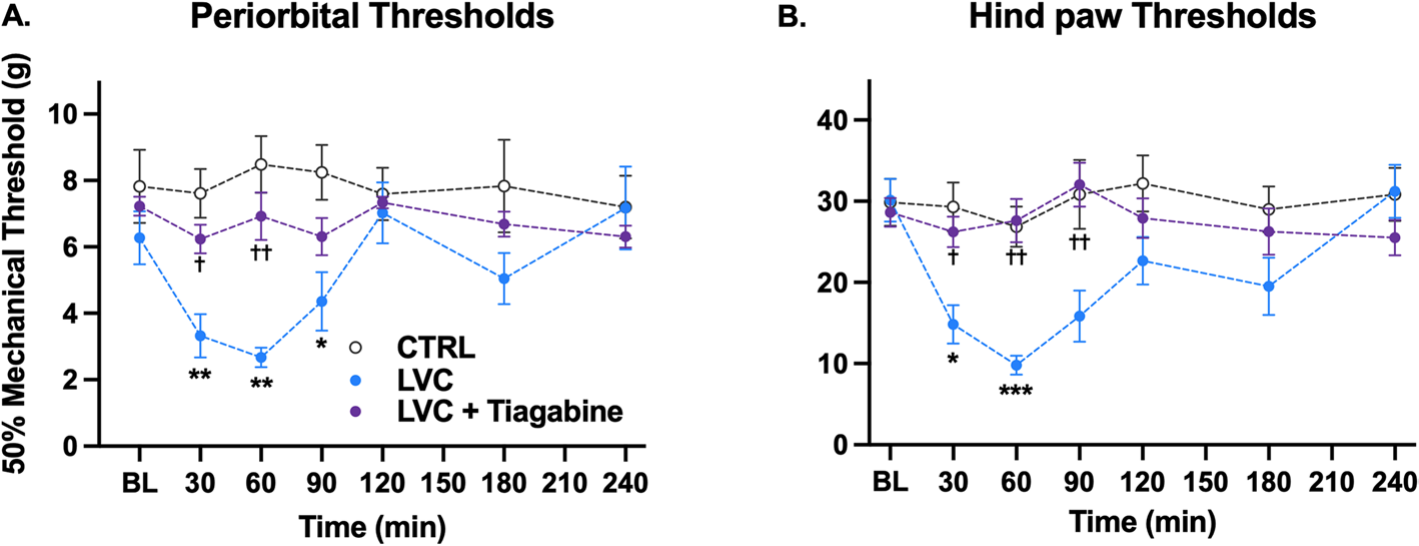
GAT-1 inhibitor tiagabine ameliorated LVC-induced periorbital and hind paw allodynia in Wistar rats. Wistar rats were injected with 5 mg/kg tiagabine and 1 mg/kg LVC. We have explored if mimicking GAT-1 overactivity in Wistar rats could prevent the mechanical threshold reduction. Tiagabine injection prevented periorbital (**A**) and hind paw (**B**) allodynia in LVC-administered Wistar rats (Sidak’s multiple comparisons two-way ANOVA, *P* < 0.05, *n* = 8 per group) (*P < 0.05 LVC vs CTRL; ** *P* < 0.01 LVC vs. CTRL; *** *P* < 0.001 LVC vs. CTRL; ✝ *P* < 0.05 LVC vs. treatment; ✝✝ *P* < 0.05 LVC vs. treatment)

At first, we wanted to evaluate the role of increased GABA concentrations in the synaptic cleft. To increase the synaptic GABA concentrations and to imitate the GAERS phenotype, we used tiagabine to block GAT-1 transporters. Tiagabine injection fully ameliorated the LVC-provoked mechanical allodynia both in periorbital and hind paw regions (F (1, 14) = 5.51, *P* < 0.05, Fig. 4A and F (1, 17) = 6.54, *P* < 0.05, Fig. 4B, respectively).

Regarding how increased GABA concentrations block the pain sensitivity response, we have hypothesized that increased GABA in the synaptic cleft should act on the postsynaptic receptors, namely GABAA and GABAB receptors. In the following experiments, we used various pharmacological interventions to delineate the receptor type further.

### GABAB receptor agonist baclofen does not mitigate acute mechanical allodynia in Wistar rats

**Fig. 5:**
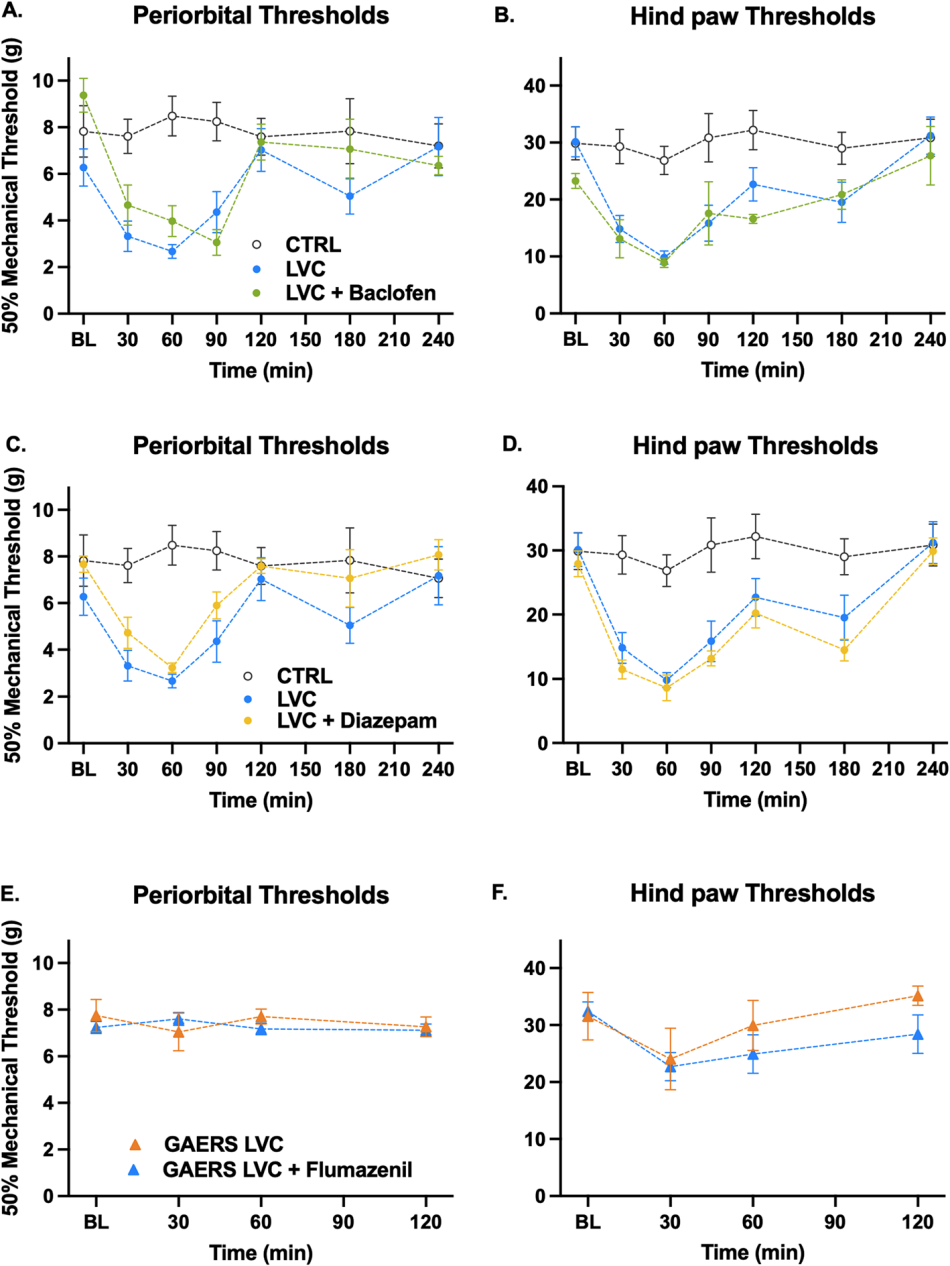
GABAB and synaptic GABAA receptors do not significantly mediate GAERS resistance to LVC and NTG. GABAB receptor agonist baclofen (2 mg/kg) (**A**, **B**) or synaptic GABAA receptor agonist diazepam (1 mg/kg) (**C**, **D**) were given to Wistar rats right after LVC administration. None blocked periorbital or hind paw allodynia in LVC-injected Wistar rats (Sidak’s multiple comparisons two-way ANOVA, *P* > 0.05, *n* = 8 per group). In line with the diazepam data, synaptic GABAA receptor antagonist flumazenil (10 mg/kg) (**E**, **F**) did not reverse the resistance when given together with LVC to GAERS (Sidak’s multiple comparisons two-way ANOVA, *P* > 0.05,* n* = 8 per group). Statistical significances between CTRL and LVC groups were not marked on the graphs where no significant treatment effect between LVC and treatment groups was detected

To assess the role of GABAB receptor overactivity, we administered baclofen and LVC to Wistar rats. Thereby, we wanted to evaluate if GABAB receptor agonism can alleviate LVC-induced pain sensitivity. Baclofen-injected rats still exhibited as much periorbital and hind paw allodynia when compared to the LVC group (F (1, 11) = 0.80, *P* > 0.05, Fig. 5A and F (1, 14) = 0.39, *P* > 0.05, Fig. 5B, respectively).

### Synaptic GABAAR agonist diazepam does not ameliorate mechanical allodynia in Wistar rats

The role of synaptic GABAA receptors was evaluated with diazepam. Diazepam administration did not prevent LVC-induced periorbital and hind paw tactile sensitivity (F (1, 10) = 1.37, *P* > 0.05, Fig. 5C and F (1, 13) = 0.41, *P* > 0.05, Fig. 5D, respectively).

### Synaptic GABAA receptor antagonist flumazenil does not negate the GAERS resistance

To further validate that synaptic GABAA receptors do not participate in GAERS resistance, we administered flumazenil with LVC to GAERS rats. Flumazenil- and LVC-injected GAERS rats did not exhibit periorbital and hind paw pain sensitivity when compared to GAERS rats (F (1, 10) = 0.06, *P* > 0.05, Fig. 5E and F (1, 10) = 0.98, *P* > 0.05, Fig. 5F, respectively).

### Extrasynaptic GABAA receptor agonists gaboxadol and muscimol block mechanical allodynia

**Fig. 6:**
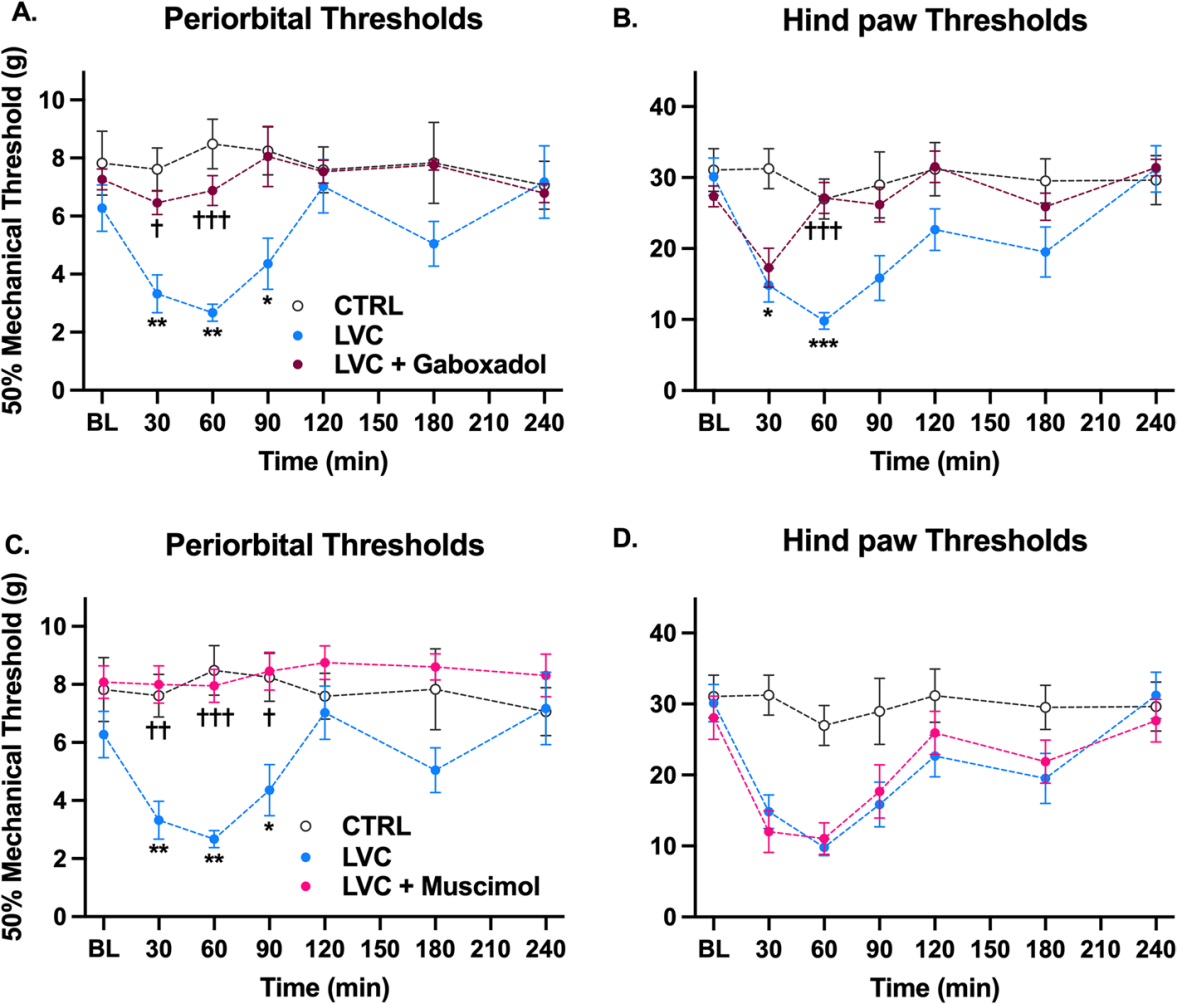
Extrasynaptic GABAA receptor agonists relieved mechanical allodynia in acute LVC-induced migraine model in Wistar rats. Gaboxadol (4 mg/kg) and muscimol (0.75 mg/kg) entirely prevented LVC-induced periorbital allodynia (**A**, **C**) (Sidak’s multiple comparisons two-way ANOVA, *P* < 0.01, *n* = 8 per group). On the other hand, gaboxadol provided only partial benefit for LVC-induced extracephalic hypersensitivity (**B**), whereas muscimol had no effect (**D**) (Sidak’s multiple comparisons two-way ANOVA, *P* < 0.05 and Sidak’s multiple comparisons two-way ANOVA, *P* > 0.05, respectively, *n* = 8 per group) (*P < 0.05 LVC vs CTRL; ** *P* < 0.01 LVC vs. CTRL; *** *P* < 0.001 LVC vs. CTRL; ✝ *P* < 0.05 LVC vs. treatment; ✝✝ *P* < 0.01 LVC vs. treatment; ✝✝✝ *P* < 0.001 LVC vs. treatment)

Finally, we wanted to explore the contribution of extrasynaptic GABAA receptors to migraine-like phenotype resistance. Gaboxadol and muscimol are well-known agonists of extrasynaptic GABAA receptors expressing delta subunit (δGABAA receptors), so we proceeded with these agents. As a result, gaboxadol fully reversed the mechanical allodynia in the periorbital area (F (1, 13) = 9.59, *P* < 0.01, Fig. 6A). It has also ameliorated the hind paw allodynia in the 60-min timepoint. Still, without any effect at the 30-min timepoint (F (1, 16) = 5.03, *P* < 0.05, Fig. 6B). On the other hand, muscimol entirely blocked the periorbital allodynia (F (1, 13) = 16.55, *P* < 0.01, Fig. 6C) with no effect on hind paw allodynia at any of the timepoints tested (F (1, 16) = 0.002, *P* > 0.05, Fig. 6D).

## Discussion

**Fig. 7:**
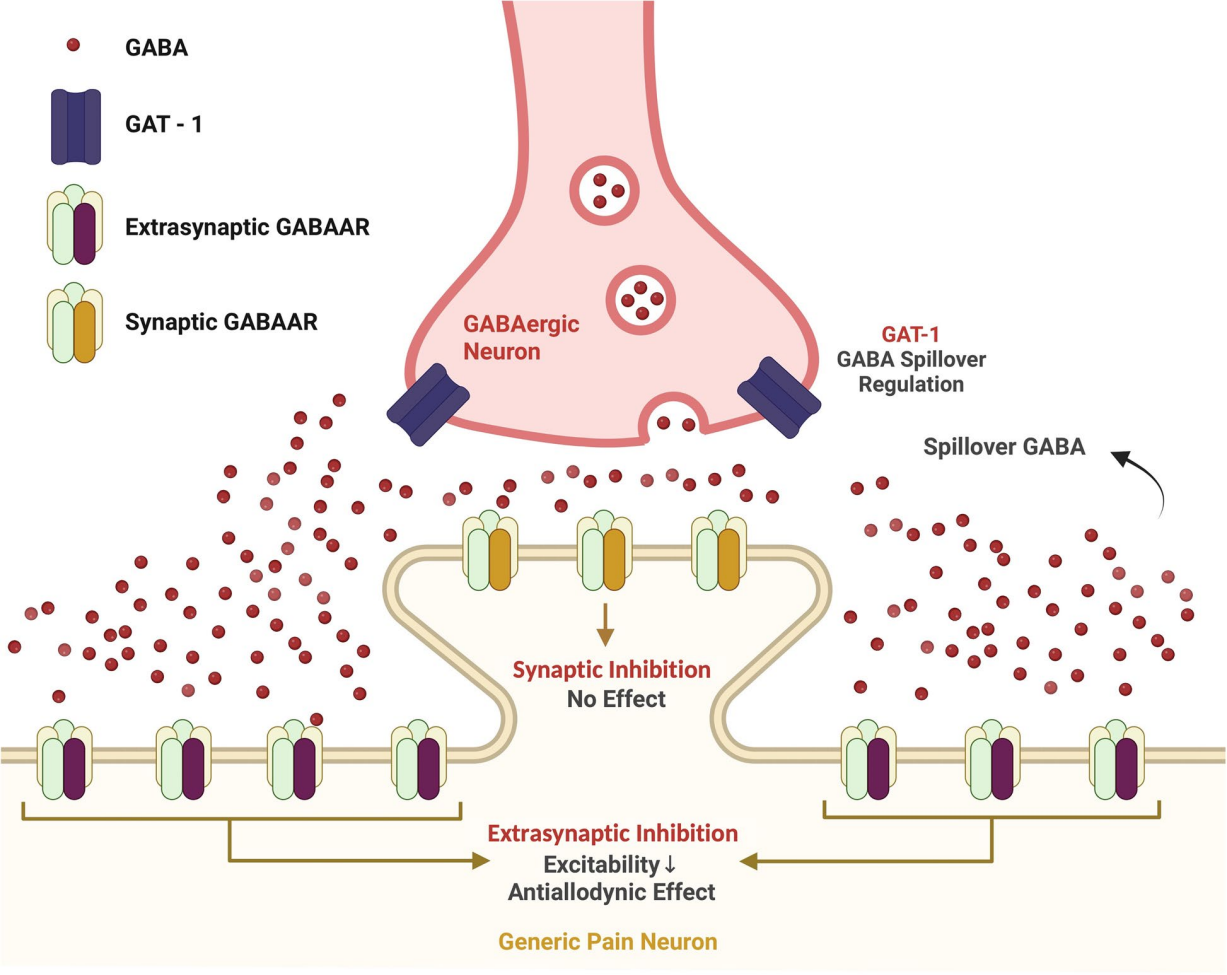
Schematic illustration of extrasynaptic δGABAA receptors in nociceptive neuron determining mechanical pain thresholds. Extrasynaptic δGABAA receptors mediate constant background inhibition on the postsynaptic nociceptive neurons, thereby modulating pain thresholds. Direct activation of δGABAA receptors by agonist drugs or indirect activation via increased spillover GABA due to GAT-1 (GABA transporter-1) inhibition may block acute migraine attacks

Our study has revealed several novel findings: GAERS rats show resistance to NTG- and LVC-provoked acute migraine models. With this, we described for the first time an animal model that is not affected by the acute injection of two well-established migraine-provoking substances, NTG and LVC. In addition, resistance was not linked to T-type channel dysfunction and the resultant paroxysmal spike-and-wave discharges, implying the involvement of another, more fundamental mechanism. In our study, we focused on the GABAergic dysfunction. Therefore, we pharmacologically imitated the GABAergic dysfunctions of GAERS in Wistar rats step-by-step. As a result, we revealed that GAT-1 blockage and δGABAA receptor agonism successfully alleviated the allodynia in LVC-injected Wistar rats. Our key findings and hypothesis are schematized in Fig. 7.

Periorbital mechanical allodynia and hind paw allodynia are frequently used to measure migraine-like phenotype in rodents [49]. We found that NTG- or LVC-administered GAERS rats were completely free from the periorbital allodynia-inducing effects of these substances at all the time points tested. Despite a slight threshold reduction in the hind paw region upon LVC administration, it did not reach statistical significance. We further verified the resistance by assessing the presence (or absence of) light aversion. Likewise, light aversion was absent in NTG- or LVC-injected GAERS rats compared to the GAERS group, which supported the mechanical threshold experiments.

One of the most well-known characteristics of GAERS rats is that they have overactive T-type calcium channels, which also drive brief spike-and-wave discharges [50]. A simultaneous interruption of consciousness, lasting 5-10 seconds, accompanies spike-and-wave discharges. These spike-and-wave discharges arise during passive wakefulness and are not in an alert state [51]. GAERS rats actively moved in the testing apparatus during behavioral experiments and exhibited exploratory behavior, signifying a fully awake state. In addition, it is already known that spike-and-wave discharges terminate upon an unexpected sensory stimulus [52]. Our preliminary electrocorticographic experiments also showed that spike-and-wave discharges were aborted by finger snapping, clapping, and von Frey filament application (data not shown). Therefore, spike-and-wave discharges (and resultant impaired consciousness) were not a likely confounding factor in our experiments. To entirely exclude any confounding effect of impaired T-type calcium channels and spike-and-wave discharges, we administered ethosuximide at 100 mg/kg to GAERS rats to 1) silence overactive T-type calcium channels, 2) inhibit resultant spike-and-wave discharges and accompanying short-lasting interruption of consciousness. Despite being used clinically, valproate was not appropriate in our experiments due to its wide range of pharmacological targets, which might confound our results. Ethosuximide is an absence epilepsy drug without therapeutic potential in migraine patients, which makes it suitable for our experiments. In our experiments, 100 mg/kg ethosuximide had successfully blocked all spike-and-wave discharges for approximately 2 hours in GAERS electrocorticographic recordings (data were not shown). Subsequently, we administered LVC to GAERS rats to see if they would exhibit increased pain sensitivity. We did not detect any allodynic responses in ethosuximide + LVC-injected GAERS rats, implying that T-type calcium channel dysfunction and spike-and-wave discharges do not contribute to migraine-like phenotype-resistance of GAERS. Notably, tiagabine, gaboxadol, baclofen, and muscimol are capable of aggravating spike-and-wave discharges at the given doses [32, 53, 54]. However, only tiagabine, gaboxadol, and muscimol imitated the GAERS resistance in Wistar rats. This further supports the notion that migraine-like phenotype resistance in GAERS is independent of spike-and-wave discharges.

GABAergic dysfunction is already an established phenomenon in GAERS. Increased tonic inhibitory mechanisms are thought to underlie the GAERS phenotype [31]. GAT-1 loss-of-function has already been demonstrated in GAERS, contributing to the increased inhibitory tonus [28]. To understand if the same mechanism is the causative factor of the resistance, we administered tiagabine to see if it can improve LVC-induced mechanical allodynia. Tiagabine entirely prevented the LVC-induced cephalic and extracephalic allodynia. The tiagabine dose in our study was 5 mg/kg, a dose considerably below 15 mg/kg, which was reported to cause impaired locomotion [55]. In another study, 4,5 mg/kg tiagabine did not alter the total distance traveled in the open field arena test [56]. On the bedside, there are only two studies that investigated the therapeutic potential of tiagabine in migraineurs [57]. In one of the studies, 92% of the patients experienced a reduction in their migraine headache frequency [58]. However, no official clinical trial has been done so far.

After observing that GAT-1 inhibition abolished LVC-induced pain withdrawal responses, we speculated that increased GABA in the synaptic cleft should have an inhibitory effect on nociceptive neurons through their post-synaptic receptors with a “spillover mechanism.” GABAB receptors are shown to be activated by the spillover of GABA [59]. Therefore, we hypothesized that overactivity of GABAB receptors, an essential mediator of inhibitory tonus, could explain the resistance [60, 61]. Within the context of migraine, baclofen was reported to reduce headache frequency, duration, and intensity in 86,2% of patients [62]. However, baclofen is not used to treat migraine headaches. The analgesic effect of baclofen in humans is very limited [63, 64]. Our study did not detect any improvement in allodynic phenotype induced by LVC in Wistar rats. We have ascertained that the baclofen dose (2 mg/kg) was the safest maximum dose, as higher doses were significantly associated with increased sedation/decreased locomotion [65, 66]. In support of our study, 2-hydroxysaclofen did not reverse the inhibitory action of GABA on thalamic neurons, implying GABAergic tonus may primarily act through extrasynaptic GABAA receptors in ventral posteromedial thalamic neurons [67]. Furthermore, it was shown that plasma protein extravasation in a substance P-induced headache model does not involve GABAB receptors [68]. The lack of an antiallodynic effect of baclofen in our study’s LVC-induced acute migraine model can be attributed to the variations in baclofen dosage. Baclofen has a dose-dependent and divergent effect profile in a tonic-clonic epilepsy model, where a low dose of baclofen (1 mg/kg) was found to exacerbate epileptic discharges via inhibition of presynaptic GABAergic neurotransmitter release [69]. Several rodent studies demonstrate baclofen’s analgesic effects [66, 70]. These studies evaluated analgesia with a hot plate test (general thermal nociception) and formalin test (inflammatory pain). On the other hand, migraine models are accepted to be “constellation” pain models, belonging neither to inflammatory nor neuropathic categories [71]. This distinction may underlie why baclofen was ineffective in our acute migraine model. A third reason is the administration route. Many studies used the intrathecal route to administer BAC, which may have a different dose-effect relationship [63, 64, 72, 73]. In our acute migraine model, we have administered 2 mg/kg baclofen through the intraperitoneal route, which is accepted to be more physiological than intrathecal administration. It should also be considered that GABAB receptor’s presence in the trigeminal pain system may not translate to antiallodynic activity. In conclusion, GABAB receptors do not alleviate allodynia induced by LVC, which posits that GABAB receptors may not play a role in GAERS resistance.

Then, we tested for synaptic GABAergic mechanisms by using diazepam and flumazenil. Diazepam is not indicated for migraine attacks or prophylaxis. Sedation and dependence are major limitations for diazepam use [74]. In our preliminary experiments, more than 1 mg/kg diazepam caused behavioral impairment (decreased vigilance and increased sedation) that was incompatible with the further experiments. A 1 mg/kg dose was still effective in behavioral models other than migraines [75]. Diazepam failed to block the LVC-induced mechanical allodynia in normal Wistar rats. To validate that synaptic GABAA receptors do not mediate the resistance in GAERS, we have also administered flumazenil and LVC to GAERS rats. Flumazenil was given subcutaneously to extend the half-life of the drug, as it has a very short half-life when given through other routes [76, 77]. We did not observe any reduction in mechanical thresholds after flumazenil and LVC administration to GAERS rats, further verifying our results. Synaptic GABAA receptors are the primary mediators of phasic inhibition [11]. Phasic inhibition is a short-lasting hyperpolarization that generates and sustains the oscillatory rhythms in neural circuits. On the other hand, tonic inhibition increases the membrane conductance, decreases the likelihood of action potential generation, and creates an input-filtering effect [11, 78]. Ataka et al. reported that tonic inhibitory currents carried more charge than phasic currents in lamina II of the spinal cord, implying tonic currents are more prominent in sensory processing [79].

Extrasynaptic δGABAA receptors are present in all regions of the peripheral and central pain matrix [19–21, 80–82]. Pyramidal neurons, interneurons, and glial cells are shown to express extrasynaptic δGABAA receptors, which contribute to tonic inhibition [83–85]. Therefore, we investigated the role of extrasynaptic δGABAA receptors. δGABAAR agonists gaboxadol and muscimol inhibited the periorbital pain response in LVC-injected Wistar rats. The dose of gaboxadol in our study was much lower than the ataxic 10 mg/kg dose [86, 87]. Muscimol dose was deliberately chosen as 0,75 mg/kg from preliminary data due to higher doses being associated with increased sedation. Gaboxadol entirely blocked LVC-induced periorbital allodynia but did not reverse the hind paw allodynia at 30 minutes. This was remarkably comparable with the GAERS pattern after the LVC administration. This pattern differs from tiagabine’s, which successfully prevented LVC-induced cephalic and extracephalic allodynia at all time points. This may suggest the contribution of different GABAergic receptors other than δGABAA receptors in the case of tiagabine. A primary reason why we did not detect such a contribution could be attributed to the fact that we conducted our experiments with doses suitable and compatible with von Frey testing. This necessitated meticulously chosen doses that would not hamper locomotion, cause sedation, or decrease vigilance. Higher doses of GABAB receptors and synaptic GABAA receptor agonists (baclofen and diazepam) might have been beneficial regarding pain phenotype. Still, these higher doses were not compatible with von Frey testing.

As for muscimol, periorbital mechanical threshold reduction was abolished entirely, yet hind paw threshold reduction was not alleviated. So far, this is the first time a divergence between periorbital and hind paw thresholds has been seen in an acute migraine model. The previous studies provide only a few insights, and further investigations are necessary to explain this phenomenon. In our research, muscimol did not alleviate hind paw allodynia, whereas gaboxadol had a partial antiallodynic effect. Although both are classified as δGABAA receptor agonists, gaboxadol also had agonistic effects on α1- and α2-containing synaptic GABAA receptors [88, 89]. On the other hand, no such information exists for muscimol, which could explain its total lack of effect on extracephalic hypersensitivity. However, both successfully blocked LVC-induced periorbital allodynia, a more direct readout of migraine-related cephalic sensitivity [44, 90]. No clinical study has evaluated the therapeutic potential of gaboxadol and muscimol in migraine headaches. The agents, their mechanisms of action, the aims, and the resultant effects are summarized in Table 2.

**Table 2:** Summary of pharmacological agents and their effects

	**Compound**	**Mechanism of Action**	**Aim**	**Antiallodynic Effect**	
**Wistar**				Periorbital	Hind paw
	Tiagabine	GABA reuptake inhibitor	Blockage of GABA transporter (GAT-1)	+++	+++
	Baclofen	GABAB receptor agonist	Activation of GABAB receptor	NE	NE
	Diazepam	Synaptic GABAA receptor agonist	Activation of synaptic GABAA receptor	NE	NE
	Gaboxadol	Extrasynaptic GABAA receptor agonist	Activation of extrasynaptic GABAA receptors	+++	+
	Muscimol	Selective extrasynaptic GABAA receptor agonist	Activation of extrasynaptic GABAA receptors	+++	NE
**GAERS**				**Mechanical Threshold Change**	
	Ethosuximide	T-type calcium channel blocker	To block spike-and-wave discharges	NE	NE
	Flumazenil	Synaptic GABAA receptor antagonist	Inhibition of synaptic GABAA receptors	NE	NE

+ sign signifies the presence of antiallodynic effect on LVC-induced mechanical threshold reduction. NE (no effect) signifies no change of mechanical thresholds following LVC administration. +++ and + show full and partial antiallodynic effect, respectively. The direction of antiallodynic effect was given with respect to vehicle groups

Our study presents some limitations. This study cannot claim to explain the full mechanism of resistance in GAERS. Rather, it introduces a novel drug target with a therapeutic potential for migraine attacks. Further studies should focus on the dose-response relationship, side effect profile, and prophylactic potential of tiagabine, gaboxadol, and muscimol within the context of migraine treatment. It is also not established how the contribution of the extrasynaptic GABAergic mechanisms relates to vascular, neuroglial, and dural aspects of migraine pathophysiology. In addition, it is still unknown whether GAERS are also resistant to chronic migraine or medication-overuse headache models, which may have a different pathogenetic origin. Additionally, we have used only male rats for the sake of comparability with the previous research. Future studies should include a female rat group as well.

In conclusion, our study introduced extrasynaptic GABAA receptors as a novel drug target within the context of migraine research and further cemented the role of tonic GABAergic inhibition on the pain matrix. We have also described for the first time a migraine phenotype-resistant rat strain, which could pave the way for the development of other novel therapeutic avenues. Future investigations are still necessary as to the machinery and the role of δGABAA receptors in pain phenotype resistance.

## Data Availability

Data will be made available upon reasonable request.

## Abbreviations

NTG: Nitroglycerin
LVC: Levcromakalim
GAERS: Genetic Absence Epilepsy of Rats Strasbourg
CTRL: Control
SEM: Standard error of the mean
GAT-1: GABA transporter 1
